# Emerging Functions of Natural IgM and Its Fc Receptor FCMR in Immune Homeostasis

**DOI:** 10.3389/fimmu.2016.00099

**Published:** 2016-03-15

**Authors:** Hongsheng Wang, John E. Coligan, Herbert C. Morse

**Affiliations:** ^1^Virology and Cellular Immunology Section, Laboratory of Immunogenetics, National Institute of Allergy and Infectious Diseases, National Institutes of Health, Rockville, MD, USA; ^2^Receptor Cell Biology Section, Laboratory of Immunogenetics, National Institute of Allergy and Infectious Diseases, National Institutes of Health, Rockville, MD, USA

**Keywords:** natural IgM, FCMR, autoimmunity, homeostasis, inflammation

## Abstract

Most natural IgM antibodies are encoded by germline Ig sequences and are produced in large quantities by both mice and humans in the absence of intentional immunization. Natural IgM are reactive with many conserved epitopes, including those shared by microorganisms and autoantigens. As a result, these antibodies play important roles in clearing intruding pathogens, as well as apoptotic/necrotic cells and otherwise damaged tissues. While natural IgM binds to target structures with low affinity due to a lack of significant selection by somatic hypermutation, its pentameric structure with 10 antigen-binding sites enables these antibodies to bind multivalent target antigens with high avidity. Opsonization of antigen complexed with IgM is mediated by cell surface Fc receptors. While the existence of Fc alpha/mu receptor has been known for some time, only recently has the Fc receptor specific for IgM (FCMR) been identified. In this review, we focus on our current understandings of how natural IgM and FCMR regulate the immune system and maintain homeostasis under physiological and pathological conditions.

## Introduction

Studies of IgG antibodies produced in response to foreign antigens have provided a wealth of information about the mechanisms involved in generating a seemingly limitless array of antigen-binding specificities by diversification of their antigen-binding domains through somatic recombination and mutation. In addition, a large number of investigations have shown that the effector functions of these antibodies are mediated through the interactions of their Fc domains with a series of isotype-restricted receptors expressed on a variety of hematopoietic cell types.

An Fc receptor specific for IgM, now termed FCMR (1), was defined only 8 years ago. Importantly, and in contrast to most secreted IgG antibodies, secreted IgM (sIgM) can be subdivided into natural and immune IgM. Natural IgM is found at equivalent levels in sera of normal and germ-free mice, and it is thought that exposure to natural antigens is responsible for its production. In addition, natural IgM is often polyreactive, whereas immune IgM is selected for antigen-specificity and is usually produced following exposure to pathogens. Because it is impossible to separate the effects of natural IgM from immune IgM when we evaluate the consequences of interactions between FCMR and an IgM molecule in non-immunized individuals; in this review, serum sIgM is taken to be synonymous to natural IgM.

Over the last several years, advances in understanding the various functions of sIgM and its interactions with FCMR have been accelerated by the generation of knockout mice that lack sIgM or FCMR. The purpose of this review is to describe new aspects of the nature and functions of the sIgM–FCMR axis.

## Natural IgM, an Overview

sIgM exists in all vertebrate species including fishes, amphibians, reptiles, birds, and mammals (2). Both mice and humans have large quantities of circulating sIgM (300–800 μg/ml for mice and 400–2300 μg/ml for humans). The serum levels of sIgM are maintained similarly in mice that are raised in pathogen-free, germ-free, or chemically defined antigen-free environments indicating that endogenous stimuli are responsible for its production (3, 4). The cellular origin of sIgM has been postulated to be predominantly B-1 cells found in the peritoneal cavity (5). However, this view has recently been revised by evidence that B-1 cells residing in the spleen and bone marrow are significant producers of sIgM (6). Other cell types including marginal zone B cells have also been implicated in the production of natural IgM (7). B-1 cells, particularly the CD5^+^ B-1a subset, belong to a stable population maintained by self-renewal independent of gut microbiomes (8, 9). B-1a cells, especially the PC1^lo^ subset (10), constantly migrate out of the peritoneum with some migrating to the spleen where they undergo activation and secret sIgM. In addition, the bone marrow is found to host a small number of CD5^−^ plasma cells originating from the peritoneum that likely contribute to long-term sIgM production (11). A thorough discussion of natural antibody-producing cells can be found in a recent elegant review (12).

Polyreactivity and autoreactivity are two prominent features found within the sIgM population. Prior studies with monoclonal natural antibodies including IgM and IgG isotypes demonstrated that a single natural IgM or IgG is capable of binding more than three apparently structurally unrelated antigens (13–15). Based on immunoadsorption experiments or immunoblotting using a panel of self-antigens, it has been estimated that 5–100% of normal mouse or human sera is autoreactive [Reviewed in Ref. (16)]. These properties of sIgM have been attributed to the germline configuration of their V region structures, characterized by enrichment of positively charged amino acids, especially arginine (17, 18). Also, compared to induced IgM antibodies, the V regions of sIgM have relatively higher frequencies of tyrosine and serine residues, which bear side-chain hydroxyl groups, allowing sIgM to bind various epitopes via ionic and hydrogen bonding (17, 18). These interactions, however, are usually of low affinity (19). Nevertheless, the polymeric binding between pentameric sIgM and a target antigen reaches a functional affinity (avidity) much higher than the intrinsic affinity (20, 21). By using the surface plasmon resonance technique to determine binding avidities, Diaw et al. analyzed five monoclonal sIgM against dissimilar autoantigens including cytoskeletal antigens and DNA. The kinetic binding constants of all five sIgM were indistinguishable from those observed for immune antibodies (22).

The biological functions of sIgM have been described to include removal of apoptotic cells, protection from infection, and tissue homeostasis (16, 23). The third function is attributed to sIgM-mediated clearance of tissue-breakdown molecules and binding to cell surface molecules on B and T cells to inhibit cell division and/or activation, thereby minimizing inflammation (16). This immunomodulatory role of sIgM has been confirmed by studies with two independently genetically engineered mouse strains lacking serum IgM (Sμ^−/−^) (24, 25). These mice exhibit abnormal B cell development, impaired antibody responses, and enhanced production of autoantibodies (see below).

## The IgM Fc Receptor, FCMR

Originally identified and termed as TOSO [encoded by Fas apoptosis inhibitory molecule 3 (FAIM3)] in 1998 (26), FCMR was recently rediscovered and characterized as an IgM-specific Fc receptor (27, 28). A consensus nomenclature for this molecule has recently been proposed as FCMR (1). FCMR is a transmembrane protein with a predicted molecular weight of ~41 KDa, but with heavily O-linked glycosylation in the extracellular domain, a mature molecule can reach ~60 KDa (26, 27, 29). The extracellular domain contains a single Ig-like domain with binding activity to the IgM Fc region, while the intracellular domain contains tyrosine residues that serve as phosphorylation sites to initiate/mediate signaling cascades. A thorough review of the molecular features of FCMR has recently been published (30).

To date, most of our understanding of the functions of FCMR comes from studies of FCMR-deficient mice. Natural mutations of FCMR in humans have not been reported. There are three independently generated *Fcmr* gene knockout strains have been reported (31–34) and two of them were characterized in detail (31, 32, 34) (Table 1). Clear differences exist among these mice, possibly due to the nature of gene targeting strategies, differing involvement of 129/Sv ES cells, extent of backcrossing to the B6 background, and husbandry environment. Readers are reminded of a recent report that revealed an astonishing “side effect” of passenger mutations of the 129 line that persists even after extensive backcrossing (35). This finding could explain why distinct knockout strains for the same gene often yield discrepant functional results.

**Table 1:** **Phenotypes of *Fcmr*^−/−^ mice**.

Reports by		Choi et al. (31)	Ouchida et al. (34)	Honjo et al. (32)	Nguyen et al. (33)
Strain		Fcmr^tm1Mak^	Fcmr^tm1Ohno^		Fcmr^tm1.2Khl^

Genetic manipulation		Deleted exons 2–8. Involving 129/Sv ES cells and backcrossing with C57BL/6 mice. The Neo gene cassette was not deleted after recombination	Deleted exons 2–4. Involving 129/Sv ES cells and backcrossing with C57BL/6 mice. The Neo gene cassette was deleted after recombination		Deleted exons 4–7. The Neo gene cassette was deleted after recombination. Pure B6 background

B cells	BM	Small pre-B↓ Immature B↓	Not changed	Not changed	NR

	Spleen	FOB↓, MZB unchanged	FOB unchanged, MZB↓, transitional B↑	MZB↓, B-1↑	B cells↓

	PerC	B-1a↑, B-2↓	Not changed	Not changed	NR

T cells		Not changed	Not changed	Not changed	Not changed
Basal Ig levels		IgG1↓, No change for other classes	IgM↑, no change for total IgG	IgM↑, IgG3↑, no change for other classes	NR

TI responses		Enhanced	Reduced	Phosphorylcholine-specific responses are enhanced	NR

TD responses		Enhanced	Reduced	Reduced	NR

Responses to infectious pathogens		NR	NR	IgM and IgG3 responses to low doses of *Streptococcus pneumoniae* are enhanced	NR

Spontaneous autoantibody production		Anti-ds-DNA and ANAs ↑	Anti-DNA, -rheumatoid factor, and ANAs ↑	Anti-DNA, -chromatin, and ANAs ↑	NR

*NR, not reported*.

## Expression of FCMR

The expression of FCMR in different cell types has been investigated at both the mRNA and protein levels by a number of investigators without complete agreement. The ImmGen database, a microarray-based public resource of mouse transcript expression (www.immgen.org), indicates a broad expression pattern of *Fcmr* among all lymphoid and myeloid cells tested, with relative expression levels ranging from ~50 in T cells, NK, DCs, myeloid, and stromal cells to ~5000 in B cells. Our quantitative PCR analyses of sorted populations revealed a similar pattern of expression, also with B cells expressing the highest levels (31). Northern blot analysis of human tissues also revealed a broad expression pattern of FCMR in lymphoid and non-lymphoid tissues (26, 28).

Fc receptor specific for IgM protein expression has been assessed in a variety of cell types using several monoclonal antibodies. Kubagawa and colleagues reported that FCMR expression was restricted to human B, T, and NK cells, and mouse B cells (27, 32). The lack of expression of mouse FCMR by non-B cells was confirmed by Ohno and colleagues (28, 34). However, Lang et al. using a different monoclonal antibody reported expression of FCMR on myeloid cells (36). On the other hand, Honjo et al. could not detect expression of exon 2 mRNA of *Fcmr* and FCMR protein in granulocytes with their monoclonal antibodies (37). Analysis of FCMR expression is complicated by the fact that it undergoes internalization after binding IgM (29). Freshly isolated tonsillar B and T cells are negative for FCMR on the cell surface; however, these cells become positive for FCMR after a brief culture with IgM-negative medium *in vitro* (27). Therefore, detection of FCMR at the cell membrane becomes problematic and ambiguous depending on the method used for study. In addition, future studies are warranted to determine whether the various anti-FCMR monoclonal antibodies recognize the same or alternative forms of FCMR expressed in different tissues.

It should be recognized that FCMR, while specific for IgM, is not the only cell surface Fc receptor capable of binding IgM. The Fcα/μ receptor (FCA/MR), encoded by the *FCA/MR* gene in humans, is an unusual Fc receptor in that it binds to two different antibody isotypes, IgA, and IgM (38). The receptor is broadly expressed in humans and mice, but with significant differences in expression patterns between the two species, particularly on hematopoietic cells (39). Both IgA and IgM cross-compete for binding to the mouse receptor suggesting a common site of interaction. Pentameric IgM does not have to contain J chain to bind the receptor (40).

A second receptor with dual specificity for IgA and IgM is the polymeric immunoglobulin receptor, PIGR. This receptor only binds polymeric IgA and IgM associated with the J chain at high affinities (41). In contrast to FCA/MR and FCMR, PIGR is expressed only on epithelial cells (42).

## Signaling Potential of FCMR

The intracellular domain of FCMR contains several tyrosine residues but lacks a commonly present immunoreceptor tyrosine-based activation motif (ITAM) and/or the immunoreceptor tyrosine-based inhibition motif (ITIM) (27). However, the FCMR cytoplasmic tail does contain an Asp–X_5_–Asp–Tyr^401^–Ile–Asn sequence that matches the recently identified immunoglobulin tail tyrosine (ITT) phosphorylation motif Glu/Asp–X_6–7_–Asp–Tyr–X–Asn present in membrane IgG (mIgG) and mIgE (43). This consensus motif is found to amplify BCR signals in class-switched memory B cells by recruiting the adaptor Grb2, thereby allowing switched memory B cells to respond more quickly and vigorously than primary B cells to secondary exposures to antigens (43, 44). Therefore, the ITT motif of FCMR could serve as a molecular platform to interact with and influence the BCR signaling pathway. In fact, cross linking FCMR with either anti-FCMR monoclonal antibodies or preformed IgM immune complexes induced phosphorylation of tyrosine and serine residues of FCMR in B cells (27) and phosphorylation of PLC-γ and Erk1/2 in NK cells (45). Our analyses also showed that FCMR positively modulates tonic BCR signaling. The basal levels of phospho-Syk in pre-B, MZ, and B-1a cells were lower in *Fcmr*^−/−^ mice than WT controls (31). Ligation of FCMR alone did not affect cellular proliferation and survival (34). However, BCR-induced proliferation and survival were reduced in the absence of FCMR (31, 34). Moreover, ligation of FCMR had no effect on LPS or anti-CD40 antibody-induced proliferation and survival (31, 34). These results argue that FCMR more closely approximates the activity of BCR than TLR or CD40 on the surface of B cells. Therefore, it is conceivable that FCMR plays a modulatory role in BCR signaling that supports survival.

## The sIgM–FCMR Axis in Regulation of Early B Cell Development

Early stages of B cell development are characterized by ordered gene expression consisting of H and L chain gene rearrangements in pro-B and pre-B cells, respectively. The expression of productive μH chains leads to assembly and expression of pre-BCRs on the surface of pre-B cells. In conjunction with the IL-7 signaling, pre-BCRs trigger clonal expansion for approximately six divisions (46) and upregulate expression of IRF4, which promotes pre-B cell dissociation from stromal cells, a critical step leading to differentiation of small pre-B cells and L chain gene rearrangement (47).

The expression levels of FCMR in early B cells gradually increases starting from pro-B to pre-B and to immature B cells (ImmGen database), similar to our assessment by qPCR (31). In the absence of FCMR, as noted in the Fcmr^tm1Mak^ strain but not the Fcmr^tm1Ohno^ strain (Table 1), the development of pre-B and immature B cells was significantly reduced (31). Remarkably, Sμ^−/−^ mice lacking sIgM exhibited similar deficiencies in pre-B and immature B cells (31, 48). Therefore, the sIgM–FCMR axis may represent a positive feedback loop promoting development and maturation of early B cells. Indeed, irradiated normal recipient mice receiving WT hematopoietic stem cells (HSCs) and sIgM-containing sera generated significantly more pre-B cells than recipients of WT HSCs and sIgM-deficient sera (31). This observation suggests that sIgM could enhance generation of pre-B and immature B cells in a FCMR-dependent fashion (31).

## The sIgM–FCMR Axis in Regulation of Late Stages of B Cell Development

Fc receptor specific for IgM deficiency in mice was associated with altered distributions of peripheral B cells. The Fcmr^tm1Mak^ strain exhibited reduced numbers of splenic follicular B cells, but the numbers of MZ B cells remained unchanged (31). In the peritoneum, the frequencies of B-1a cells were increased while B-2 cells in this strain were decreased (31). In contrast, the Fcmr^tm1Ohno^ strain exhibited reduced numbers of MZ B cells, increased transitional B cells and B-1 cells in the spleen, and no change in peritoneal B cells (32, 34) (Table 1). The differences in distribution of B cells between the two strains are also associated with differences between the strains in basal levels of serum immunoglobulins in naive mice (Table 1) (31, 32, 34, 48).

Our parallel assessments of Sμ^−/−^ and Fcmr^tm1Mak^ mice revealed strikingly similar changes in peripheral B cells: reduced numbers of FO but increased numbers of MZ B cells in the spleen, and increased numbers of B-1a cells but reduced B-2 cells in the peritoneum (31). A second Sμ^−/−^ strain also exhibited expansion of MZ and B-1a cells and reduction in FO B cells (25). These data agree with the conclusion that the sIgM–FCMR axis may promote FO B cell development. Regarding B-1 cells, Nguyen et al. recently reported that peritoneal CD5^+^ B cells in Sμ^−/−^ mice were not “regular” B-1a cells but exhibited characteristics of “anergic” B cells (48). They further showed that the lack of sIgM in Sμ^−/−^ mice not only altered B cell numbers (reduced FO and expanded MZ B cells), confirming our analyses (31), but also fundamentally altered the B cell repertoire (the usage of V_H_ genes) and selection (48). More importantly, administration of sIgM into Sμ^−/−^ mice reversed the MZ/FO B cell ratio and mostly restored normal B cell development (48, 49). These results strongly suggest that the sIgM–FCMR axis does indeed modulate differentiation of peripheral B cells.

## The sIgM–FCMR Axis in Regulation of T-Independent Immune Responses

Immunization of the Fcmr^tm1Mak^ strain with NP-LPS stimulated increased levels of plasma cell development and secretion of IgM antibodies (31). This data can be explained by increased numbers of peritoneal B-1a cells in *Fcmr*^−^*^*/*^*^−^ mice as B-1a cells are the primary responders to NP-LPS challenge in this setting (31). Immunization of Fcmr^tm1Mak^ mice with NP-FICOLL, which predominantly activates MZ B cells, resulted in moderate increases in plasma cells without a significant increase in sIgM, which seems to correlate with relatively normal numbers of MZ B cells in this strain (31). In contrast, studies of the Fcmr^tm1Ohno^ strain yielded opposing results, i.e., reduced production of sIgM and IgG following NP-FICOLL immunization (34) (Table 1). This discrepancy could be due to the fact that the MZ B cell compartment is reduced in the Fcmr^tm1Ohno^ strain but remains unchanged (if not slightly expanded) in the Fcmr^tm1Mak^ strain. By using a complicated antigen, *Streptococcus pneumoniae* (R36A), Honjo et al. showed increased IgM and IgG3 responses to the carbohydrate phosphorylcholine (PC), epitope of the pathogen, in Fcmr^tm1Ohno^ mice (32).

Previous studies in Sμ^−/−^ mice revealed enhanced production of IgG2a antibodies following immunization with NP-FICOLL (50). Similar findings were reported in another Sμ^−/−^ strain (25). Taken together, these results suggest that the sIgM–FCMR pathway may negatively regulate TI immune responses likely through modulating the sensitivity of responding B cells.

## The sIgM–FCMR Axis in Regulation of T-Dependent Immune Responses

Studies of TD immune responses in Fcmr^tm1Ohno^ and Fcmr^tm1Mak^ mice have also yielded conflicting results. Our analyses in the Fcmr^tm1Mak^ strain revealed a moderate increase in primary IgM but not of IgG2b responses following immunization with NP-KLH/alum (31). During a recall immune response, FCMR-deficient mice produced significantly more germinal centers and plasma cells but only exhibited subtle differences in overall serum antibody levels (31). In contrast, following a similar immunization protocol, Ouchida et al. and Honjo et al. reported that the Fcmr^tm1Ohno^ mice developed generally decreased TD immune responses (32, 34) (Table 1). Given the fact that the antibody responses elicited in both strains were relatively moderate, we believe that the role of FCMR in TD immune responses may be limited.

Previous studies of Sμ^−/−^ mice indicated that sIgM is required to elicit optimal TD immune responses because in the absence of sIgM, Sμ^−/−^ mice produced significantly lower levels of IgG1 anti-NP antibodies, and antibody affinity maturation was also delayed (25). However, injection of sIgM into Sμ^−/−^ mice before immunization increased NP-specific IgG1 responses (25). These results suggest that sIgM could augment TD humoral responses, but whether this was mediated by sIgM-mediated antigen processing/presentation, or FCMR-mediated signaling that renders B cells hyperresponsive to activation signals, or both, remains to be determined.

## The sIgM–FCMR Axis Suppresses Development of Autoimmunity

Spontaneous production of increased levels of autoreactive antibodies, including anti-DNA and anti-nuclear antibodies, was detected in both FCMR-deficient strains (Table 1). Surprisingly, the existence of these autoantibodies in FCMR-deficient mice was insufficient to induce pathology, e.g., glomerular damage common to lupus-like diseases. Introducing *Fcmr* deficiency onto the autoimmune-prone B6.MRL Fas^lpr/lpr^ background accelerated development of autoreactive antibodies but still had no effect on severity of renal pathology and function or overall survival (51). These results suggest that FCMR acts to inhibit activation of non-pathogenic autoreactive B cells.

Absence of sIgM in Sμ^−/−^ mice was also associated with accelerated development of IgG autoantibodies (48, 52, 53). Similar to FCMR-deficient mice, both Sμ^−/−^ strains spontaneously generated IgG anti-DNA autoantibodies (52, 53). Immune complex deposition in the kidneys was observed in a small proportion of mice (53). When Sμ^−/−^ mice were bred to MRL^lpr/lpr^ mice, the progeny exhibited accelerated development of IgG autoantibodies and autoimmune disease (52). Moreover, adoptive transfer of Sμ^−/−^ BM cells into recipient mice containing normal levels of sIgM abrogated anti-nuclear antibody development (48). Thus, sIgM and FCMR may negatively regulate autoimmunity through the sIgM–FCMR pathway not withstanding that sIgM may also modulate autoimmune responses through FCMR-independent mechanisms, such as complement fixation, immune complex uptake, and antigen presentation (23).

In addition to the functions of FCMR in the above lupus model, Lang et al. studied the roles of FCMR in an experimental autoimmune encephalomyelitis (EAE) model and demonstrated that Fcmr^tm1Mak^ mice were resistant to EAE (54). While changes in expression of IL-17 in T cells were not observed in this report (54), a more recent study using single cell RNA-sequencing in isolated Th17 cells identified a strong correlation between expression of FCMR and the Th17 cytokine signature (55). Indeed, FCMR-deficient T cells fail to produce IL-17A upon stimulation (55). IL-17A is a critical driving cytokine for EAE (56). Thus, FCMR may promote pathogenesis of Th17-mediated diseases.

## Does FCMR Play a Role in Controlling Infection?

While sIgM has long been postulated to play an important role as first line in defense against infectious agents (2), there are also indications that FCMR may play a role during inflammatory responses to infection. Honjo et al. immunized Fcmr^tm1Ohno^ mice with a live attenuated strain of *S. pneumoniae* (R36A) to examine antigen-specific immune responses against the bacterial PC and protein determinants (32). While the FCMR-deficient mice successfully elicited anti-PC antibody responses, they failed to generate anti-protein antibody responses (32). This result suggests that FCMR has discrete roles in B cells (possibly coupled with specificity of the BCR) in responding to protein and non-protein determinants of live pathogens.

Lang et al. performed wide range analyses of the Fcmr^tm1Mak^ strain infected with *Listeria* (36). FCMR-deficient mice were resistant to LPS-induced septic shock and failed to control Listeria infection. This phenotype is associated with decreased systemic production of IFN-γ, IL-12, and IL-6 in FCMR-deficient mice (36). While most of the changes in this model were attributed to absence of FCMR in phagocytes, including monocytes, macrophages, and granulocytes (36), the expression of FCMR by these myeloid cells was questioned by Honjo et al. who failed to detect expression of exon 2 of *Fcmr* by PCR and protein by FACS in granulocytes (37). More recently, Lang et al. have reported that FCMR is required for development and function of inflammatory dendritic cells (iDCs) (57). FCMR mutant mice were deficient for iDCs in the liver and thereby failed to recruit and activate CD8^+^ T cells to clear lymphocytic choriomeningitis virus (57). Because the expression levels of FCMR transcripts in granulocytes and DCs are about 100-fold lower than in B cells (ImmGen database) and it remains unknown whether infection could upregulate expression of FCMR in granulocytes and DCs, future studies are warranted to determine the molecular mechanisms of FCMR action in non-B cells in response to infection.

## Concluding Remarks

It is becoming broadly accepted that sIgM has protective functions in defense against infection and is important for maintaining tissue homeostasis. Earlier studies have revealed that sIgM binds to infectious agents, as well as altered self-antigens, and that sIgM-antigen complexes activate the complement cascade and initiate inflammatory responses. The recent identification of FCMR has provided new insights into the mechanisms of sIgM function *in vivo*. While evidence of the beneficial effects of sIgM on survival have dominated the literature, it is worth noting that sIgM can also play pathogenic roles under certain circumstances. For example, natural IgM is critical for development of inflammation and damage in a model of ischemia–reperfusion injury (58–61). In this case, sIgM binds to damaged tissue and activates the complement cascade causing massive inflammatory responses. Blocking sIgM binding to modified self-antigens with an anti-annexin IV single-chain antibody (scFv) significantly reduced graft inflammation and injury (58). Recently, Panzer et al. reported that injection of sIgM into B-cell-deficient μMT mice induced albuminuria in a complement-induced glomerular disease model (62). These observations raise a note of caution regarding postulated application of intravenous IgM (IVIgM) (63), a homolog of intravenous IgG (IVIg), to the treatment of autoimmune and inflammatory diseases. More recently, Brenner et al. reported that a FCMR-Fc fusion protein comprising the extracellular domain of FCMR exhibited therapeutic effects by inhibiting MOG-induced neuroinflammatory responses in an EAE model (54). An anti-FCMR monoclonal antibody has also been shown to be beneficial in experimental malaria infection (64). Future studies are warranted to determine whether blockade of FCMR could be a therapeutic approach to treat autoimmune and inflammatory diseases.

## Author Contributions

All authors listed, have made substantial, direct, and intellectual contribution to the work, and approved it for publication.

## Conflict of Interest Statement

The authors declare that the research was conducted in the absence of any commercial or financial relationships that could be construed as a potential conflict of interest.
